# A comparison of immunogenicity and protective immunity against experimental plague by intranasal and/or combined with oral immunization of mice with attenuated *Salmonella* serovar Typhimurium expressing secreted *Yersinia pestis* F1 and V antigen

**DOI:** 10.1111/j.1574-695X.2007.00280.x

**Published:** 2007-07-19

**Authors:** Wen-Tssann Liu, Hui-Ling Hsu, Chung-Chih Liang, Chuan-Chang Chuang, Huang-Chi Lin, Yu-Tien Liu

**Affiliations:** 1 Institute of Preventive Medicine, National Defence Medical Center Taipei, Taiwan; 2 Institute of Microbiology and Immunology, National Defence Medical Center Taipei, Taiwan

**Keywords:** intranasal immunization, *Salmonella* vaccine, *Yersinia pestis* F1, V antigen

## Abstract

We investigated the relative immunogenicity and protective efficacy of recombinant X85MF1 and X85V strains of ΔcyaΔcrpΔasd-attenuated *Salmonella* Typhimurium expressing, respectively, secreted *Yersinia pestis* F1 and V antigens, following intranasal (i.n.) or i.n. combined with oral immunization for a mouse model. A single i.n. dose of 10^8^ CFU of X85MF1 or X85V induced appreciable serum F1- or V-specific IgG titres, although oral immunization did not. Mice i.n. immunized three times (i.n. × 3) with *Salmonella* achieved the most substantial F1/V-specific IgG titres, as compared with corresponding titres for an oral-primed, i.n.-boosted (twice; oral-i.n. × 2) immunization regimen. The level of V-specific IgG was significantly greater than that of F1-specific IgG (*P*<0.001). Analysis of the IgG antibodies subclasses revealed comparable levels of V-specific Th-2-type IgG1 and Th-1-type IgG2a, and a predominance of F1-specific Th-1-type IgG2a antibodies. In mice immunized intranasally, X85V stimulated a greater IL-10-secreting-cell response in the lungs than did X85MF1, but impaired the induction of gamma-interferon-secreting cells. A program of i.n. × 3 and/or oral-i.n. × 2 immunization with X85V provided levels of protection against a subsequent lethal challenge with *Y. pestis*, of, respectively, 60% and 20%, whereas 80% protection was provided following the same immunization but with X85MF1.

## Introduction

Plague is a zoonotic disease caused by the Gram-negative bacterium *Yersinia pestis* (Perry & Fetherstone, 1997). Two distinct forms of plague exist, namely bubonic and pneumonic plague (Perry & Fetherstone, 1997). Bubonic plague, a disease characterized by massively swollen lymph nodes, is transmitted primarily by the bite of an infected flea and the congestion of bacteria-contaminated foods. Pneumonic plague occurs as the result of the progress of bubonic plague, from direct contact between infected humans (animals) by means of droplets expelled during coughing, or from inhalation of aerosolized *Y. pestis* from a biological weapon. It usually has a short incubation period of 2–3 days, and a fairly high mortality rate if the condition remains untreated (Perry & Fetherstone, 1997; Titball & Leary, 1998; Inglesby *et al.*, 2000).

Although several antibiotics such as tetracycline and amoxicillin have previously been used for front-line prophylaxis for, as well as treatment of, plague infection (Russell *et al.*, 1996; Galimand *et al.*, 1997), over time the relative effectiveness of such a treatment regimen has been challenged as a consequence of the ever-increasing emergence of multi-drug-resistant *Y. pestis* isolates (Guiyoule *et al.*, 2001). There is thus clearly a pressing need for the development of an appropriate plague vaccine. The currently licensed plague vaccine is killed whole-cell vaccine; however, it has been shown that its effectiveness is unsatisfactory for pneumonic plague in a mouse model, and its use does feature some local and systematic side effects (Marshall *et al.*, 1974; Meyer *et al.*, 1974). In recent years, international research efforts appear to have focused on the use of recombinant technology to design a new vaccine candidate for immunization for such diseases, with the principal focus being directed towards *Y. pestis* F1 and LcrV (V) antigens (Titball & Williamson, 2001). The F1 (17.5-kDa) polypeptide is a specific virulence factor of *Y. pestis*, and is transcribed and secreted into the bacteria surface by means of the Caf system, which consists of Caf1A as an anchor, Caf1M as a chaperone, and certain Caf1 (F1) structural proteins (Galyov *et al.*, 1991; Karlyshev *et al.*, 1992). The F1 antigen is a capsular-like protein that has been previously associated with the antiphagocytosis of *Y. pestis* within mouse macrophage-like J774.A.1 cells (Pettersson *et al.*, 1999; Du *et al.*, 2002). The LcrV antigen is common to all three varieties of human pathogenic *Yersiniae*. It is also involved in controlling the secretion of an array of *Yersinia* outer proteins (Yops) from bacteria cytoplasm (Price *et al.*, 1991), and would appear to be quite closely associated with immune suppression (Nakajima & Brubaker, 1993; Nakajima *et al.*, 1995; Nedialkov *et al.*, 1997; Welkos *et al.*, 1998). Both the F1 and V antigens have been tested successfully as potential plague-vaccine antigens, either alone or in combination with appropriate adjuvants, following parenteral or intranasal immunization, enabling the production of protective immunity against plague for an experimental animal model (Leary *et al.*, 1995; Williamson *et al.*, 1995; Anderson *et al.*, 1996; Heath *et al.*, 1998; Jones *et al.*, 2006).

Live attenuated *Salmonella* vaccine strains have been used as carriers of heterologous antigen(s) from bacteria, viruses and parasites (Cardenas & Clements, 1992). Following oral administration, *Salmonella* has been shown to be capable of stimulating systemic antibody and cell-mediated immunity (Chatfield *et al.*, 1994; Hormaeche *et al.*, 1995). Similarly, immunization through the intranasal route with *Salmonella* has been shown to be as efficient as, and even superior to, the oral route in inducing mucosal and systemic immune responses (Hopkins *et al.*, 1995; Pasetti *et al.*, 2000; Capozzo *et al.*, 2004). Both these approaches have also been tested in *Salmonella*-based vaccine, and successfully induced protective immunity against plague in a mouse infection model (Morton *et al.*, 2004). Conventionally, a *Salmonella* vaccine strain contains a plasmid-based expression vector, which encodes the heterologous antigen(s) of interest, and an antibiotic-resistance selection marker that is used, after addition of the corresponding antibiotic, for plasmid maintenance. The use of such *Salmonella* strains has been discouraged because of concerns over safety regarding use in humans, and because of concerns regarding cost-effectiveness, as it is necessary to produce large quantities of antibiotics by large-scale fermentation for production of the bacteria as inoculate (Hagg *et al.*, 2004; Verch & Pan, 2004).

The attenuated *S. enterica* serovar Typhimurium strain x8501 harbours deletion mutations in cya and crp, defective in the synthesis of the adenylate cyclase and cyclic AMP receptor, and asd, which encodes the aspartate β-semialdehyde dehydrogenase (Asd), an essential enzyme for cell-wall biosynthesis (Nayak *et al.*, 1998). This Asd auxotrophic mutant was unable to grow in complex medium without supplementation with diaminopimelic acid (DAP), a bacteria amino acid not found in eukaryotes, but, after trans-complementation with an Asd^+^ plasmid, the mutant's growth was restored (Gálan *et al.*, 1990). Hence, only Asd^+^ plasmid-carrying cells can grow in DAP-free medium, making the Asd^−^*Salmonella* strain dependent on the plasmid maintenance, owing to the balanced lethal relationship between vector and host systems (Nakayama *et al.*, 1988; Gálan & Sansonetti, 1996). Recently, a multicopy, stable Asd^+^ antigen-expressing vector, pAY3493, has been specially designed to express the secreted recombinant pneumococcal surface protein (rPspA) antigen by means of the fusion of the β-lactamase signal sequence in an Asd^−^*Salmonella* vaccine strain (Kang *et al.*, 2002). This particular strain of *S. typhimurium* x8501 (pYA3493) has been demonstrated to be capable of inducing an enhanced immune response to rPspA.

In our study, we describe the construction of a ΔcyaΔcrpΔasd-attenuated strain of *S. typhimurium* expressing either the *Y. pestis* F1 or V antigens as a candidate plague vaccine. Furthermore, we undertook an assessment of the immune response of test mice and of the protective efficacy of these *Salmonella*-derived vaccines against plague following mucosal immunization with such vaccines within a mouse infection model.

## Materials and methods

### Bacterial strains, plasmid and growth condition

*Salmonella enterica* serovar Typhimurium strain x8501 is aΔcrp-28ΔasdA16 mutant, and pYA3493 is an Asd^+^ and pBRori vector containing β-lactamase signal sequence (Kang *et al.*, 2002). The Asd^−^ mutant x8501 strain was cultivated at 37°C in Luria–Bertani (LB) broth or Luria agar (L-agar) containing diaminopimelic acid (DAP, 50 μg mL^−1^; Sigma Chemical Co., St Louis, MO). The recombinant *Escherichia coli* DH5/pUC18-CafAMF1 (EC1853F strain) harbouring the Caf operon used to produce the F1 antigen was constructed as described previously (Titball *et al.*, 1997).

### Construction of recombinant plasmid expressing secreted F1 or V antigen

Standard DNA-manipulation techniques, such as PCR, agarose gel electrophoresis, restriction-enzyme digestion, ligation and bacterial transformations, were performed according to the procedures described previously (Sambrook *et al.*, 1989). Plasmid pYA3495 was obtained by replacing the multiple cloning site of pYA3493 with M oligonucleotides (5′-GAATTCGAGCTCGGTACCCGGGGATCCAAGCTT-3′) containing EcoRI/SacI/KpnI/BamHI/HindIII sites. Isolation of the pMT1 and pYV1 plasmids, endogenous plasmids from *Y. pestis* EV76S, was performed according to the method described in Kado & Liu (1981). Construction of the F1-expressing plasmid was by means of a two-step process. First, a specific DNA fragment encoding Caf1 was amplified by PCR from pPMT with primers PF1B (5′-GTTCCGGGATCCATGAAAAAAATCAGTTCCGTT-3′, underlined for the BamH1 linker region) and PF1H (5′-GTTCCGAAGCTTTTATTGGTTAGATACGGTTAC-3′, underlined for the HindIII linker region), cleaved by BamH1/HindIII, and cloned into pYA3495 to yield pYA3495F1. Second, a PCR-derived product containing Caf1M and its promoter region amplified from pMT1 with primers PM11E (5′-GTTGAATTCTATCAAAATTAGCTATTTGCGCAA-3′, underlined for the EcoR1 linker region) and PM12BG (5′-GTTAGATCTAAATATTACCTCTATCGAATAATC-3′, underlined for the BglII linker region) was cleaved with EcoRI/BglII, and this product was cloned into pYA3495F1 with EcoRI/BamHI cleavage in order to yield the expression plasmid pYA3495MF1. Similarly, the V-encoding gene was amplified from pYV with the primers PV11H (5′-GTTCCGAAGCTTTCATTTACCAGACGTGTCATC-3′) and PV12B (5′-GTTCCGGGATCCATGATTAGAGCCTACGAACAA-3′), cleaved by EcoRI/BglII, and cloned into the EcoRI/BamHI site of pYA3495 in order to yield pYA3495V. The resulting plasmids from pYA3495MF1 and pYA3495V were transformed into the *S. typhimurium* strain x8501 by electroporation (Bio-Rad, Hercules, CA) in order to raise the *Salmonella* X85MF1 and X85V strains, respectively.

### *In vitro* and *in vivo* expression of F1 and V antigens

For *in vitro* expression of the F1 and V antigens, bacterial strains X85MF1 and X85V were cultured in LB broth at 37°C incorporating 200-r.p.m. shaking for a period of 24 h. Subsequent to harvesting the cultures by centrifugation (7000 ***g***, 10 min), the soluble fraction was concentrated using a low-molecular-weight cut-off filter (10 kDa, Amicon Ultra, Millipore Co. Bedford, MA), and the cell-pellet fraction was subjected to protein extraction using BugBuster Protein Extraction reagent (Novagen Inc., Madison, WI) following the procedures recommended by the manufacturer. Expression of F1 and V was confirmed by sodium dodecyl sulfate polyacrylamide gel electrophoresis (SDS-PAGE) separation of each sample and analysed by immunoblotting using anti-F1/V-specific monoclonal antibodies (F1-4B5-3 and V-4H-10, laboratory collection). The relevant reactive polypeptides were detected by means of the chemiluminescent substrate super signal (Pierce, Rockford, IL). Expression of F1 and V was also examined within a mouse macrophage-like cell line J774.A1 (ATCC TIB 67) following a series of protocols as described previously (Morton *et al.*, 2004). Briefly, the J774.A1 cell line was cultured in a six-well slide-chamber containing Dulbecco minimal essential medium (DMEM; GIBCO BRL, Grand Island, NY) supplemented with 10% fetal calf serum (FCS; GIBCO BRL). Mouse macrophage cells were then infected with *Salmonella* X85MF1, X85V or the control x8501/pYA3493 strain, as appropriate, at a multiplicity of infection of 50 : 1, and subsequently incubated at 37°C in a humidified atmosphere containing 5% CO_2_-in-air for a period of 1 h. Following this, the extracellular bacteria present were killed by the use of gentamicin (100 μg mL^−1^), and the cells were then incubated for a further period of 24 h. Subsequent to incubation, macrophage cells were washed in PBS, fixed with 4% formaldehyde, and incubated with the mouse anti-F1 or -V antigen antibody for a period of 1 h. Following PBS washing, the macrophage cells were incubated with a cocktail solution containing Alex-Red conjugated goat anti-mouse IgG and phallodin fluorescein isothiocyanate (FITC)-conjugated rabbit anti-mouse antibody (Molecular Probes Inc., Eugene, OR) in order to detect the specific F1/V antigen-antibody and to probe the tublin in order to examine the morphology of J774A.1 cells. Finally, coverslips were mounted onto the slides and slides were visualized using a fluorescence confocal microscope (Leica DM IRE2; Zmst-Leitz-Strasse, Wetzlar. Germany).

### Immunization of mice with *Salmonella*

Groups of five, 8-week-old female BALB/c mice were purchased from the Animal Center of Taiwan University. Test mice were inoculated with recombinant *S. enterica* serovar Typhimurium vaccine X85MF1, X85V or x8501/pYA3495 (1–3 × 10^8^ CFU in a 20-μL PBS aliquot) via an intranasal pathway in the right naires using a 20 μL Gilson pipette, the mice being allowed to inhale the bacteria as previously described (Pasetti *et al.*, 2000). Alternatively, mice were orally inoculated with an equivalent dose of *Salmonella* suspended in 0.5 mL of PBS. The immunization regimens consisted of either i.n. or oral-priming with two subsequent i.n. boostings (at 2-week intervals for each boost) with *Salmonella* vaccine.

### Measurement of serum antibody

Mice were bled via the tail vein on day 14 subsequent to each immunization. Serum samples were assayed for IgG antibody titres specific for F1 and V by means of an enzyme-linked immunosorbent assay (ELISA) (Sambrook *et al.*, 1989). The *Y. pestis* F1 antigen derived from the EC 1853F strain was purified to homogeneity by size-exclusion chromatography as described previously (Andrews *et al.*, 1996). The purified His_6_-V antigen was prepared from recombinant *E. coli* DH5/pQE30-lcrV expressing *Y. pestis* V using an Ni-NTA superflow column by FPLC (fast-performance liquid chromatography; Amersham Pharmacia Biotech, Uppsala, Sweden). Polystyrene 96-well microtitre plates were coated with purified F1 or V (100 ng well^−1^). Serum samples obtained from mice in the same experimental group were pooled and diluted serially. A 100-μL volume of diluted samples was added to each well of the microtitre plate in duplicate and incubated for 2 h at 37°C. Following this, the horseradish peroxidase (HRP)-conjugated goat-anti mouse IgG (H+L) or IgG subclass (Southern Biotechnology, BD) was added, it functioning as detector antibody for a specific serum antibody, and the substrates *o*-Phenyldiamine dihydrochloride (OPD) and H_2_O_2_ were added. Solution absorbance was measured at 490 nm (A_490 nm_) using an ELISA reader, and antibody concentrations were calculated by interpolating regression-corrected OD_490 nm_ values for the diluted sample according to the dilution produced by a known concentration (9.8∼312 ng mL^−1^) of purified IgG, IgG1 or IgG2a antibodies that had bound to a microplate that had been pre-coated with goat anti-mouse Ig.

### Evaluation of antibody- and cytokine-secreting cell numbers in the spleen and lung

The modified enzyme-linked immunospot (ELISPOT; Eyles *et al.*, 2004) assay was performed to enumerate the antibody-secreting cells (ASC) against F1 and V in the spleen and lung of immunized and naïve mice in order to determine the potential cellular immune response to immunization. Briefly, Immobilon-P fixed (Multi Screen-IP, Millipore) 96-well microtitre plates were coated with 100 μg mL^−1^ of recombinant F1 or V antigen (10 μg mL^−1^ in PBS), incubated overnight at 4°C, and then blocked with 200 μL of complete culture medium (RPMI 1640 containing 10% FBS and 1% penicillin–streptomycin) for a period of 1 h at room temperature. Following this, 100 μL of spleen-cell suspension (derived from individual mice), at various densities ranging from 1 × 10^5^ to 2 × 10^6^ mL^−1^ in complete culture medium, was added to antigen-fixed 96-well microtitre plates in triplicate, and the plates were placed in a humidified (5% CO_2_-in-air) incubator at 37°C for 24 h. The plates were washed, and then incubated with aviden-HRP-conjugated MAb against mouse Ig (IgG, A; KPL, Gaithersburg, MD) at 37°C for a period of 1 h, followed by washing three times with PBS containing 0.1% Tween-20. Finally, 100 μL of 3-amino-9-ethylcarbazole substrate (Sigma) was added to each well, and the plates were incubated at room temperature for various periods ranging from 15 to 60 min, following which reactions were stopped by washing wells once with distilled water. Reddish-brown coloured ‘spots’ were enumerated and scored for Ab-forming cells under a dissecting microscope. In order to assess the number of cytokine-secreting cells, equal volumes of the F1 or V protein (40 μg mL^−1^) were added to the cells in test wells of plates, and incubated for 24–48 h under normal conditions for *in vitro* stimulation. Subsequent to washing, the cells were coincubated with 2 μg mL^−1^ biotinylated anti-IL-10 and/or IFN-γ antibody (Pharmingen) and examined microscopically, as described previously (Eyles *et al.*, 2004). The data were expressed as the mean number of cytokine- or antibody-secreting cells±SE/10^5∼6^ cells.

### *Yersinia pestis* challenge

*Yersinia pestis* (Yokohama-R strain) cultured in brain/heart infusion agar (Difco, Sparks, MD) plates for 48 h was collected and prepared for subsequent inoculation using PBS. Bacterial counts were enumerated by plating the dilute of inoculate on a Congo-Red plate as previously described (Andrews *et al.*, 1996). Groups of eight BALB/c mice were individually intraperitoneally (i.p.) injected with the virulent *Y. pestis*, with doses ranging from 2 × 10^4^ to 2 CFU at 10-fold dilutions. The median lethal dose (LD_50_) end-point was determined to be at about 15 CFU according to the Reed–Muench method (Welkos & O'Brien, 1994). Two weeks subsequent to final immunization, i.e. on day 43 after primary immunization, mice were challenged i.p. with 1–2 × 10^3^ CFU (100 × LD_50_) of *Y. pestis* in a 0.5 mL PBS aliquot. The challenge studies were conducted in a biosafety level-3 containment facility, following the standard operating procedures for the facility.

### Statistical analysis

Statistical analysis was performed using the computer program microsoft excel Ver. 6.0. Levels of significance of difference in antibody response between the tested groups were determined by means of a two-tailed Student's *t*-test. Fisher's exact probability test was performed to compare the mortality for the immunized group of mice with the corresponding controls. A value of *P*≤0.05 was considered a statistically significant difference between tested datasets.

## Results

### Secreted expression of F1 and V antigens

**Fig. 1:**
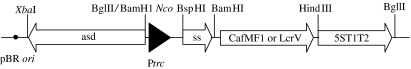
Plasmids used for the expression of *Yersinia pestis* F1 or V antigen for an attenuated *Salmonella* typhimurium strain. The *Y. pestis* CafMF1- or LcrV-encoding gene was cloned into the expression vector pYA3495 (see ‘Materials and methods’ section) downstream of the Bla-secretion system. This Bla-secretion system consisted of the β-lactamase signal sequence and 12 amino-acid residues of the *N*-terminus of the mature β-lactamase signal sequence, which derived from plasmid pBR322, and which was expressed under the control of the Ptac promoter. 5ST1T2 is a transcriptional terminator.

**Fig. 2:**
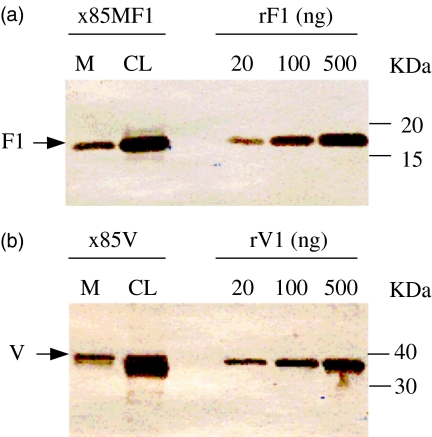
Comparison of *Yersinia pestis* F1 and V antigen expression *in vitro* in a *Salmonella* strain. The overnight cultures of (a) X85MF1 and (b) X85V bacterial strains were fractionated, separated by SDS-PAGE, and then probed, separately, with mAb against F1 (4B5-3) and V (4H-10). Samples equivalent to 5 × 10^7^ CFU of X85MF1 and 10^7^ CFU of X85V from overnight culture of bacteria lysate were loaded into SDS-PAGE. Lanes codes: M, the supernatant fraction collected from centrifugation following overnight culture; CL, total cell lysate. Various amounts of purified rF1 and V protein (in ng) are shown at the top of the figure, and molecular-weight markers (in kDa) are indicated on the right-hand side of the figure.

It has previously been reported that the Caf1M chaperone protein is required for successful secretion expression of the hIL-1β-Caf1 chimera protein on cells of recombinant *E. coli* (Galyov *et al.*, 1991). We thus elected to incorporate Caf1M-Caf1 into the expression vector pYA3495. The structure of the corresponding plasmids for expression of rF1 or rV antigens is depicted in Fig. 1. The lysate derived from various fractions of the *S. typhimurium* x8501/pAY95MF1 (X85MF1) and x8501/pAY95V (X85V) cultures were separated by SDS-PAGE, and specific proteins were examined by immunoblot assay. A fairly large polypeptide was observed from the culture supernatant as well as from the cell-pellet fraction, it being *c*. 17 and 37 kDa, respectively, for X85MF1 and X85V (Fig. 2). The majority of rF1 and rV was located in the cell-pellet fraction, and *c*. 2–5% of the total rF1/V was present in the culture supernatants. This result indicates that rF1/V is highly expressed and is actively secreted via the secretion machinery from the construct. Following this, the levels of rF1 and rV expression in X85MF1 and X85V strains were quantified using an alkaline phosphatase-conjugated ELISA procedure. Samples of X85MF1 strain were shown to express *c*. 50 μg rF1 per 10^9^ CFU cells. In contrast, the expression level for the X85V strain reached as substantial a level as 250–300 μg rV per 10^9^ CFU cells. Immunoblotting of known quantities of purified proteins also confirmed a greater yield of V than of F1 antigen for X85V and X85MF1 samples.

### Expression of F1 and V within macrophage-like J774.1A cells

**Fig. 3:**
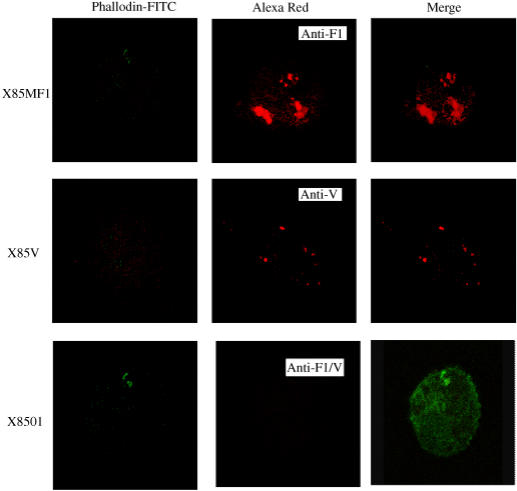
Confocal immunofluorescent microscopy images of *Yersinia pestis* F1- and V-antigen expression for X85MF1 and X85V bacterial strains within mouse macrophage-like J774 A1 cells. The J774 A.1 cells were infected with the *Salmonella* vaccine strain, and the intracellular bacteria were probed with anti-F1 or anti-V mAb and then detected with Alex-Red conjugated anti-mouse IgG (red). The cell morphology, as characterized by the cytoskeleton, was observed by Phllodin-FITC-conjugated antitubulin antibody (green).

In order to investigate F1/V expression *in vivo*, *Salmonella* bacteria were inoculated into cultured J774.1A cells, and the presence of rF1/V within macrophage cells was subsequently examined by confocal microscopy of immunofluorescently labelled X85MF1 and X85V. The results revealed that F1/V antigens were actively expressed inside the J774.1A cells, as indicated by the presence of positively fluorescent images of X85MF1- and/or X85V-infected cells, but this was not the case for the cells infected with *Salmonella* containing vector alone (X8501; Fig. 3).

### IgG antibody responses to F1 and V antigens

**Fig. 4:**
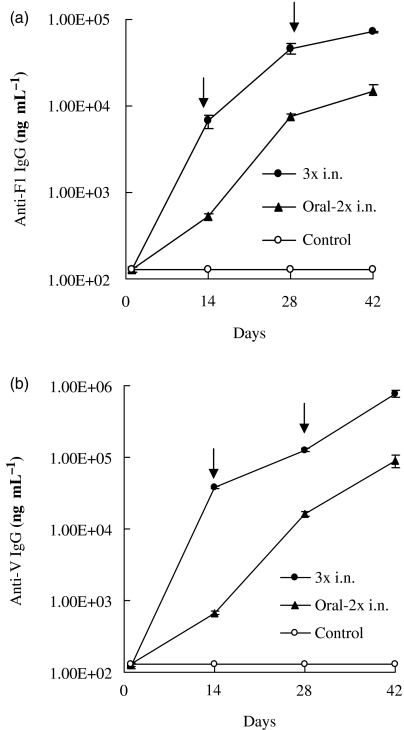
Total F1/V-specific IgG responses in pooled sera collected on days 14, 28 and 42 following i.n. × 3 or oral-i.n. × 2 immunization regimens with the *Salmonella* (a) X85MF1 and (b) X85V vaccine strains. The arrow indicates the time point, on, respectively, days 15 and 29 subsequent to primary immunization, at which each i.n. boost was administered for the specific vaccination regimen. Error bars indicate SD.

Groups of BALB/c mice that had undergone a series of two oral administrations of 10^8^ CFU of *S. typhimurium* x85MF1 and/or x85V on day 0 and day 14 did not reveal any induction of significant serum levels of IgG antibody for either F1 or V antigen (data not shown). Thus, the test mice were primed by the oral or i.n. route and boosted by i.n. immunization on days 14 and 28 of the experiment in order to attempt to assess the immunological response to the *Salmonella* vaccine, the sera of these immunized mice being collected on days 14, 28 and 42 after primary immunization. The level of serum anti-F1/V IgG titres elicited in immunized mice was found to be differently dependent upon the specific immunization regimen undertaken (Fig. 4a and b). In both the i.n. × 3 and oral-i.n. × 2 immunization regimens, *Salmonella* induced the anti-F1/V titres to a significantly greater level than was the case for the control mice. Furthermore, the i.n. prime–boost immunization regimen with either X85MF1- or X85V-produced vaccine induced a significantly greater level of anti-F1/V titres compared with the corresponding levels for the oral-i.n. × 2 prime–boost regimen (*P*<0.001). The level of test-mouse serum anti-F1/V titre was elevated following the i.n. boost, with a noted two- to sixfold increase in levels of antibody titre for each booster, irrespective of the route of priming. The highest level of serum anti-F1/V titre that was achieved followed three i.n. doses of vaccine. Interestingly, levels of serum anti-V titres elicited by such immunization regimens were found to be significantly greater than was the case for corresponding anti-F1 IgG titres, this being the case at all dosing levels investigated (*P*<0.001), whereas such an immunization regimen with *Salmonella* induced only a low, but detectable, level of F1/V-specific serum IgA (data not shown).

### IgG antibody subclass responses to F1 and V antigens

**Fig. 5:**
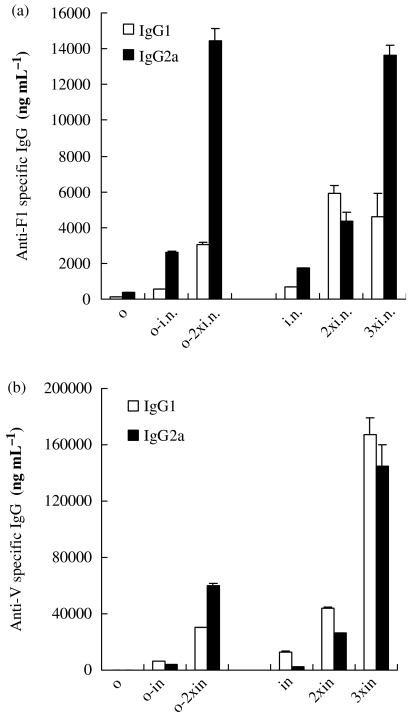
F1 and/or V antigen-specific IgG1 and IgG2a responses in sera collected on day 14 after the delivery of a dose of *Salmonella* (a) X85MF1 and (b) X85V, following i.n. × 3 and/or oral-i.n. × 2 prime–boost immunization regimens. Error bars indicate SD.

Sera prepared on day 14 subsequent to mice having been primed or boosted with either *Salmonella* vaccine were further analysed to ascertain the serum IgG1: IgG2a subclass ratio as an indirect assessment of the T helper-cell response bias (Mosmann & Coffman, 1989). The results revealed that the IgG1/IgG2a profiles developed in test mice appeared to differ depending on the vaccine antigen involved. Mice i.n. × 3 or oral-2 × i.n. immunized with X85MF1 tended to experience a Th1-type response, as evidenced by a predominant F1-specific IgG2a response to immunization. By contrast, a mixed Th1/Th2-type response, as indicated by similar levels of IgG1 and IgG2a being present in sera, developed following the same immunization regimen with X85V (Fig. 5a and b).

### Mucosal and cell-mediated immune responses induced by *Salmonella* x85MF1 and x85V vaccines

**Fig. 6:**
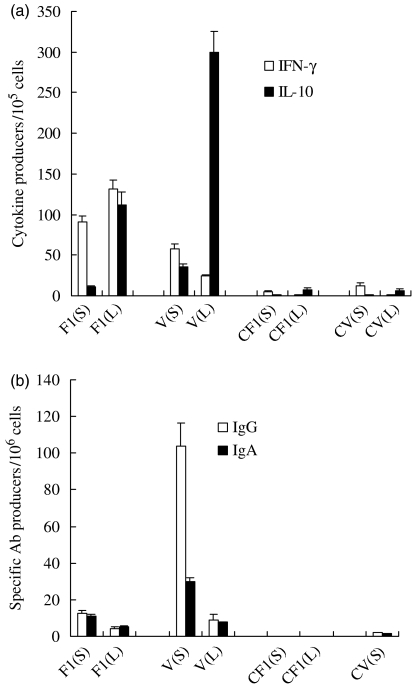
IgG-, A- and cytokine-producing cells in the spleen and lung of immunized mice. Mice were i.n. × 3-immunized with *Salmonella* X85MF1 (F1) and/or X85V (V), following which spleen (S) and lung (L) cells were examined for the production of F1/V antigen-specific antibody (a) and cytokine-secreting cells (b) by ELISPOT assay. Error bars indicate SD.

As vaccination through the intranasal route features the potential to induce mucosal protective immunity (Huang *et al.*, 2001), we investigated IgG and IgA antibody-secreting cells (ASC) in the lung tissue of test mice. The frequency of the presence of mucosal and systemic ASC following i.n. × 3 immunization with X85MF1/V was also determined. The F1/V-specific IgG ASC in the lung of test animals was shown to be fairly low when compared with those ASC detected in the spleen; however, F1/V-specific IgA ASC was hardly ever seen within lung tissue. In order to test whether mice i.n. × 3 immunized with X85MF1/V developed any cell-mediated immune response to the vaccine antigen, the presence of F1/V-specific IFN-γ- and IL-10-secreting cells was determined in the spleen and lung. A greater number of F1-specific than of V-specific IFN-secreting cells was detected both in the spleen and the lung. By contrast, the frequency of V-specific IL-10-secreting cells was significantly greater than that of F1-specific IL-10-secreting cells (Fig. 6a and b). Furthermore, stronger F1/V-specific IFN-γand IL-10 cytokine responses were elicited for the lung tissue than for spleen tissue following the i.n. × 3 immunization regimen with *Salmonella* vaccine.

### Protection of immunized mice against lethal challenge with *Y. pestis*

**Fig. 7:**
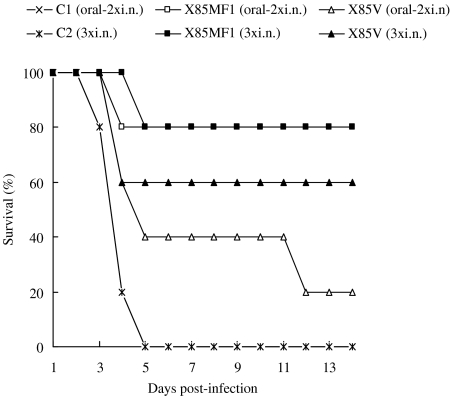
Protection against *Yersinia pestis* challenge following mucosal prime–boost immunization with X85MF1 and X85V. Two weeks after the last immunization following i.n. or oral − i.n. × 2 administration with *Salmonella* vaccine, mice were i.p. challenged with 2 × 10^3^ CFU of *Y. pestis*, and individual test-mouse survival was recorded daily for a period of 2 weeks.

Two weeks following oral-i.n. × 2 or i.n. × 3 immunization with *Salmonella* (X85MF1 or X85V), mice were i.p. challenged with 2 × 10^3^ CFU of *Y. pestis*, and subsequent test-animal survival was recorded daily for the subsequent 14-day period. All the mice immunized with *Salmonella* x8501/pYA3495 (control group) died by day 5 post-immunization. Mice orally primed and i.n. boosted twice (oral-i.n. × 2) and/or i.n. × 3 immunized with X85V experienced, respectively, 20% and 60% protection against a lethal dose of *Y. pestis* challenge. This contrasts with a corresponding figure of 80% protection provided by the same immunization regimen but using X85MF1 (Fig. 7).

## Discussion

The use of the *Salmonella* vaccine strain as a delivery system for the guest antigen(s) takes advantage of a variety of prevailing factors, namely the relative ease of antigen preparation, the possibility of vaccine self-administration, the use of a noninvasive method of immunization for vaccine development, and the reasonably cost-effective nature of such an immunization modality. All of these features could facilitate the wide-spread use of such a vaccine. In the present study, we used a mouse model to investigate the immunological responses, at both systemic and mucosal levels, to administration of the recombinant *Salmonella typhimurium* strain expressing either the *Y. pestis* F1 or V antigens by two distinct routes of mucosal immunization. These constructs of X85MF1 and X85V proved to be fairly stable *in vitro* following 40–50 generations of bacterial culture in antibiotic-free conditions, whereas following i.n. and/or oral administration to test mice the constructs appeared to diminish in persistence in test-animal spleens and lungs when compared with the control strain (x8501/pYA3495) over 5 days. For example, fewer than 5 CFU of vaccine organisms were observed to be present in the spleen or liver at 24 h postadministration of *Salmonella* x85MF1/V strains, compared with 100–200 CFU per liver or spleen for the control strain, indicating that the vaccine strains seemed to impair colonization. Although the F1 and V antigens were highly expressed and efficiently secreted by *Salmonella* X85MF1 and X85V *in vitro*, at 37°C in Luria agar only the V antigen, and not the F1 antigen, was detected on the cell surface of the bacteria, as deduced by immunofluorescent microscopy imagery of X85MF1/V probed with specific mAb (data not shown). Such a combination of results is probably associated with the absence of the Caf1A anchor protein in the Caf1M-Caf1-secretion system of the expressing vector, owing, presumably, to the essential role of Caf1A for surface localization of Caf1 (Zavialov *et al.*, 2001). To the best of our knowledge, however, at the time of writing it is unknown whether F1 secretion is independently controlled by the Caf1M-Caf1-secretion system or by the interaction of F1 with the lactamase signal sequence. Expression of F1 and V antigens was also clearly detected within vaccine-infected mouse macrophage-like J774A.1 cells (Fig. 3). No ctytotoxic effect upon cultured cells was elicited by infection with X85MF1 and/or X85V strains.

Animals that were administered *Salmonella* x85MF1/V by an i.n. or an oral route did not reveal any sign of sickness during the study. The serum antibody response of test animals was significantly induced by a single i.n. dose of *Salmonella*, although the corresponding oral immunization did not elicit such a result. The reason(s) for this outcome is unclear at present; it may be because the oral *Salmonella* vaccine fails to express the plague antigen stably (Dunstan *et al.*, 2003). The most pronounced level of serum IgG noted herein was achieved for test animals following three i.n. doses of *Salmonella*: the level appeared to be statistically significantly greater than the corresponding value for the oral-primed and i.n.-boosted (twice) immunization regimen using the same vaccine. Immunization with *Salmonella* stimulated a strong V-specific IgG response (mean antibody concentration log_10_ 4.96–5.9 ng mL^−1^), a response clearly greater than the corresponding F1-specific IgG antibody response (log_10_ 4.17–4.85 ng mL^−1^). Such an outcome may possibly be related to the greater level of V antigen expression, as compared with F1 expression, that occurred within *Salmonella*. Moreover, this outcome may also be related to V antigen, but not F1 antigen, expression on the cell surface of the *Salmonella* bacterium. These two features that in concert appeared to enhance the antibody responses of immunized test animals, whereas in contrast all the immunized mice revealed the induction of rather low levels of serum F1/V-specific IgA (data not shown).

The dominant species of the F1-specific IgG subclass is IgG2a (Fig. 5a), the presence of this immunoglobulin being indicative of Th1-type immunity. This result would appear to be consistent with the results of a number of previous studies using *Salmonella*-delivered antigen (Leary *et al.*, 1997; Bullifent *et al.*, 2000; Morton *et al.*, 2004). Similar to the results of Garmory *et al.* (2003), immunization with X85V appeared to induce different levels of V-specific IgG1 and IgG2a, but there was not much variation in the respective levels elicited by i.n. × 3 and oral-i.n. × 2 immunization (Fig. 5b). We speculated that this discrepancy in IgG-subclass profile between the F1 and the V antibody responses may have been associated with differences in the localization of expressed antigen for X85MF1 and X85V. Although the challenge route, strain and dose of virulent *Y. pestis* used in this study differed from those used in a number of previous studies (Morton *et al.*, 2004), the relative protective efficacy afforded to test mice by vaccination with X85MF1 was comparable with that reported for intranasal immunization with *S. typhi* vaccine expressing F1 (Garmory *et al.*, 2003; Morton *et al.*, 2004). It would thus appear that the relative efficacy of X85V vaccination is superior to the reported effect of using orally presented V-antibody-produced *Salmonella* vaccine (Garmory *et al.*, 2003), although the former would appear to produce results that are inferior to that of vaccination with the DNA vaccine expressing V-tPA (human plasminogen activator signal sequence) fusion protein (Wang *et al.*, 2004).

A number of related studies have indicated that antibody-mediated immunity is important for protection against plague (Titball *et al.*, 1997; Williamson *et al.*, 1999). Concurring with this observation, our results have revealed fairly high levels of antibody responses to immunization of test mice with *Salmonella*-F1/V vaccine, an outcome that would appear to provide better protection for mice counter-challenged with *Y. pestis* than would be expected to be the case for low levels of antibody responses to F1/V, suggesting that the F1- or V-specific IgG antibody response is positively correlated with the protective efficacy of vaccination of test mice with X85MF1 and/or X85V (Figs 4 and 7). Surprisingly, both the i.n. × 3- and oral -i.n. × 2-immunized mice experienced a similar level (80%) of protection against plague, and for these two immunization modalities only a subtle difference in time to death was seen, despite the elicited anti-F1 IgG antibody responses being significantly different. Nevertheless, when the same immunized mice were challenged with *Y. pestis* on day 56 subsequent to primary immunization, 80% (i.n. × 3) and 60% (oral-i.n. × 2) of mice survived such challenging (unpublished results). Furthermore, when we compared the antibody response on day 56 with the corresponding anti-F1 IgG titres determined on day 42 postimmunization we noted that there was a concomitant two- to threefold increase in the serum anti-F1 IgG antibody titres for the i.n. × 3 group, whereas a slight decline in the serum anti-F1 IgG titres for animals from the oral-i.n. × 2 group was seen (data not shown). Such a result suggests that an intranasal prime–boost immunization regimen using X85MF1/V is more likely to elicit a persistently sustained high level of antibody response than would appear to be the case for an orally primed i.n.-boosted (twice) immunization regimen; however, such so-elicited immune responses do not appear to provide full protection against i.p. challenge with *Y. pestis* for test mice. Hence, it would appear that some enhancement of the immune response elicited by immunization with the X85MF1 and X85V vaccines is necessary for improvement of the antiplague efficacy. Strategies such as heterologous prime–boost immunization regimens (Londono-Arcila *et al.*, 2002; Glynn *et al.*, 2005), *in vivo* inducible gene-expression techniques, and the incorporation of an immunity-modulating agent into the protective antigen are strategies that have been developed recently and that have been used to improve the relative immunogenicity of *Salmonella* vaccine (Atkins *et al.*, 2006); they may pave the way for the future use of X85MF1/V to this end. Furthermore, it would also appear to be possible to introduce, either alone or in combination, the F1/V antigen-expressing vector into the *S. typhi* vaccine strain as a candidate antiplague vaccine in order to test the vaccine further for subsequent human use. A recent study demonstrated that oral delivery of *Salmonella*-(F1+V) antigens elicits both F1- and V-specific antibody responses and confers enhanced antiplague efficacy to treated mice as compared with either F1 or V responses alone, emphasizing the potential application of combined (F1+V) immunization (Yang *et al.*, 2007).

The induced F1-specific IgG antibody response was shown to be significantly less pronounced than the V-specific antibody response in the serum of the immunized mice (*P*<0.001) (Fig. 4). Furthermore, the deemed mechanism of action of X85MF1 appeared to confirm the ability of the vaccine to confer better protection aganist plague for recipient animals than what appeared to be the case for X85V vaccination, this probably being the result of the functionally different virulence roles of F1 and V antigens in plague pathogenesis (Price *et al.*, 1991; Du *et al.*, 2002). In addition, the discrepancy in protective efficacy between the two agents may be attributable to the different cell-mediated response produced by the administration of X85MF1 and X85V, at both systemic and mucosal levels, as evidenced by the presence of F1/V-specific IgG IL-10- and IFN-γ-secreting cells in the spleen and lung of i.n.-immunized mice (Fig. 6). In particular, X85V vaccine appeared to induce high levels of IL-10 postvaccination, but its relative antiplague efficacy was somewhat impaired as regards the production of IFN-γcompared with X85MF1 (Fig. 6). V-mediated IL-10 secretion is thought to be involved in plague pathogenicity by preventing the release, to serum, of proinflammatory cytokines, such as TNF-α and IFN-γ, postinfection, and by subsequently suppressing the innate immune responses to plague infection (Nakajima & Brubaker, 1993; Brubaker 2003). Furthermore, certain proinflammatory cytokines have been shown to provide protective efficacy against *Y. pestis*. For example, passive immunization with TNF-α and IFN-γ has been shown to protect mice from plague infection (Nakajima & Brubaker, 1993), and the relative antiplague efficacy of F1- and V-based vaccine has been demonstrated to be reduced for Stat-4-deficient mice, animals which are typically deficient in the production of type-1 cytokine responses (Elvin & Williamson, 2004). The presumably high levels of IL-10 release following vaccination do not appear to be desirable for effective plague vaccination. Recently, a study using a V variant lacking amino-acid residues 271–300 revealed that such a vaccine elicited uncompromised protective efficacy while significantly reducing the induction of IL-10, an outcome that re-enforces the abovementioned notion (Overheim *et al.*, 2005). Together, these results appear to imply that the cell-mediated, as well as the humoral, immune responses elicited by *Salmonella* vaccine administration play an important role as regards protection against plague for vaccinated test animals.

In conclusion, the present study demonstrates that i.n. immunization with the X85MF1 and X85V bacterial strains, unlike oral immunization with the same strains, induces a strong antibody response in recipient test mice. Furthermore, these vaccines, which probably elicit different profiles of immune responses, independently reveal immunological correlation with the protection against plague as afforded to vaccine-recipient test mice. In addition, X85MF1 appears to demonstrate a greater potency than X85V in terms of the overall antiplague protective efficacy of such prime–boost immunization regimens. Our findings indicate that those *Salmonella* strains expressing F1 and/or V antigen are able to induce a profound immune response for recipient animals following i.n. vaccination, or following i.n. vaccination combined with delivery of vaccine via other routes, and that such types of vaccination are able to provide suitable protection for test animals from the deadly challenges of *Y. pestis* infection, thus providing a broad perspective for enhanced plague-vaccine development.

## Statement

Re-use of this article is permitted in accordance with the Creative Commons Deed, Attribution 2.5, which does not permit commercial exploitation.
